# Experimental Research Progress of Seismic Metamaterials: Structural Configurations, Attenuation Mechanisms, and Engineering Prospects

**DOI:** 10.3390/ma19132812

**Published:** 2026-07-02

**Authors:** Xinchao Zhang, Wei Liu, Qingfan Shi

**Affiliations:** 1 Key Laboratory of Building Collapse Mechanism and Disaster Prevention, China Earthquake Administration, Institute of Disaster Prevention, Sanhe 065201, China; zxc@cidp.edu.cn; 2 School of Physics, Beijing Institute of Technology, Beijing 100081, China

**Keywords:** seismic metamaterials, wave attenuation, local resonance, Bragg scattering, field experiments, scaled-down experiments

## Abstract

Seismic metamaterials (SMs) have emerged as a novel wave-control strategy for earthquake-resistant engineering, offering the potential to manipulate seismic waves via artificially designed periodic/resonant structures. Field and laboratory experiments are critical to bridge theoretical predictions and engineering practice, yet a systematic synthesis focusing on experimental progress remains lacking. This review systematically classifies SMs into buried (BSMs), above-surface (ASMs), and partially embedded (PESMs) configurations, summarizing their structural designs, attenuation mechanisms, experimental performance, and key limitations. Results show that SMs can achieve >70% attenuation in the 0–50 Hz seismic band, with buried periodic barriers reaching 99.7% energy blocking and forest-like ASMs achieving 93–99% Rayleigh wave reduction. PESMs exhibit superior adaptability to shallow soils, with bandgaps concentrated in 1.5–14.5 Hz (building-sensitive range). Current experiments have advanced from single mechanisms to multi-mechanism synergy and from specialized materials to conventional concrete/steel. However, critical gaps remain: scaling-induced deviations, poor complex-geology adaptability, lack of long-term durability, and insufficient multi-waveform control. Finally, we propose a 3–10-year engineering roadmap and outline future directions: multi-waveform regulation, soil–metamaterial dynamic matching, durability design, and full-scale intelligent upgrades. This work aims to provide a critical experimental reference for the practical deployment of SMs.

## 1. Introduction

Earthquakes, as catastrophic natural hazards, generate long-wavelength, high-energy seismic waves that pose persistent threats to built environments. Traditional seismic isolation strategies, which depend on structural plastic deformation, are inherently passive and prone to failure under extreme ground motions, limiting their reliability for modern infrastructure [1,2,3,4,5,6,7,8,9,10,11]. In recent decades, the concept of metamaterials, originating from condensed matter physics, has been extended to seismic engineering, giving rise to SMs as an innovative wave-control paradigm [11,12,13,14,15,16,17,18]. By engineering periodic or resonant substructures, SMs exploit Bragg scattering and local resonance to form frequency bandgaps, enabling targeted attenuation of seismic waves and offering an active alternative to conventional mitigation methods [11,12,13,14,15,16,17,18].

Theoretical and numerical investigations have laid the foundational framework for SM design. Early analytical and numerical studies confirmed that periodic arrangements can tailor seismic wave propagation [19,20]. However, most theoretical models oversimplify soil conditions, assuming homogeneous, linear elastic media while neglecting stratification, saturation, and nonlinear soil–structure interaction—factors that induce notable discrepancies between predictions and real-world performance [21]. Similarly, numerical simulations rely heavily on idealized boundaries and simplified dynamic behaviors, often failing to capture full-scale complexity and potentially underestimating structural vulnerabilities. As a result, experimental validation remains indispensable to bridge theory and engineering reality [22,23].

Experimental research stands as the core link translating SM concepts into practical applications. Current experimental efforts span laboratory-scale models and full-scale field tests, each with distinct advantages and limitations. Laboratory experiments enable controlled parametric studies at low cost but are constrained by scaling effects; field experiments reflect authentic geological and loading conditions but are limited by site access and resource intensity. Structurally, SMs can be categorized into BSMs, ASMs, and PESMs, defined by installation depth and configuration. Mechanistically, attenuation is governed primarily by local resonance (low frequency, 1–10 Hz) and Bragg scattering (mid–high frequency, 20–50 Hz), supplemented by emerging mechanisms such as inertial amplification and negative Poisson’s ratio effects to broaden effective bandwidth [24].

Despite growing experimental progress, existing studies remain fragmented, and current reviews often focus predominantly on theoretical principles or scattered case summaries, lacking a systematic, critical synthesis of experimental advances [2,3,4,5,6,7,8]. Critical gaps persist across the literature: most experimental investigations focus narrowly on Rayleigh waves, with limited attention to Love and body waves; scaling-induced deviations and real-world geological adaptability are insufficiently quantified; long-term durability and engineering scalability are underaddressed; and comparative analysis of different SM configurations remains limited [25]. Additionally, recent advances in nonstationary stochastic modeling and topological metamaterial design have yet to be systematically integrated into experimental reviews [26,27].

To address these gaps, this review provides a comprehensive, construction-oriented synthesis of SM experimental research. We classify SMs into BSMs, ASMs, and PESMs based on burial depth and installation form—a practically motivated framework absent in prior reviews. We systematically analyze structural configurations, attenuation mechanisms, experimental performance, and key limitations across laboratory and field studies, with a focus on critical comparisons of reliability, scalability, and engineering feasibility. Unlike descriptive catalogs, this work emphasizes critical evaluation of experimental data, highlights unresolved challenges, and proposes a phased engineering roadmap. By integrating recent advances, this review aims to deliver a practical, systematic reference for future SM research and real-world deployment.

## 2. Experimental Research on Buried Seismic Metamaterials (BSMs)

### 2.1. Laboratory Experiments

BSMs regulate seismic wave propagation through underground periodic or resonant structures. Laboratory scaled-down experiments are crucial for connecting theoretical simulations and engineering applications, allowing the verification of bandgap characteristics, wave regulation mechanisms, and seismic mitigation effects via similarity principles while avoiding the high costs and site limitations of full-scale tests. This section systematically summarizes the laboratory scaled-down experimental research on BSMs over the past decade, classified by their core structures and regulation mechanisms.

#### 2.1.1. Local Resonance-Dominated BSMs

The local resonance mechanism dissipates seismic energy through the “mass-spring” resonance of subwavelength units (size ≈ λ/10), making it suitable for ultra-low frequency (≤10 Hz) control. This represents a mainstream BSM route. Key performance is summarized in Table 1.

**Figure 1:**
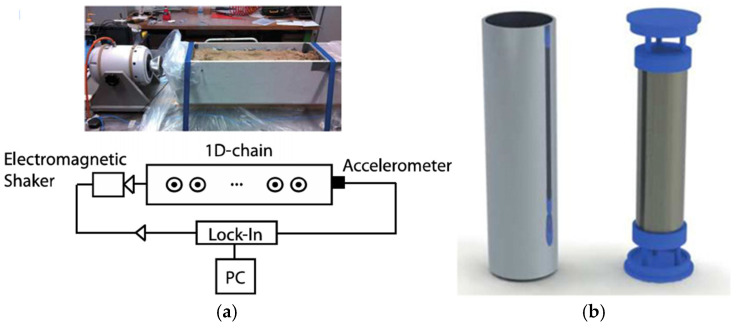
(**a**) Test setup including shaker and sand box; (**b**) scaled resonator. The connecting springs are highlighted in blue [29].

**Figure 2:**
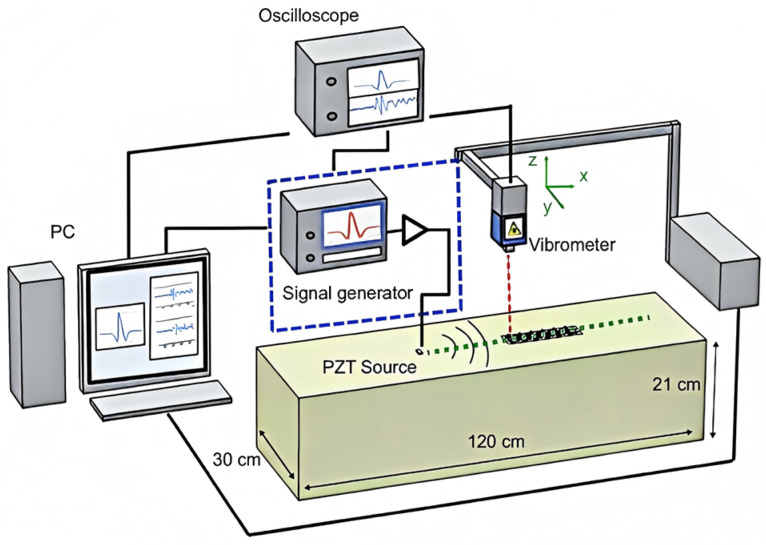
Experimental measurement setup for seismic metamaterial [35]. Reprinted with permission under CC BY 4.0 license.

(1) **Negative Poisson’s Ratio Pile-Type Metamaterials** [28]: This structure adopted a composite pile unit with a “concrete core-TPU30A negative Poisson’s ratio buffer layer-thin shell,” featuring a negative Poisson’s ratio of approximately −0.72, arranged in a 2 × 4 array. The prototype has a lattice constant of 3 m, scaled down to 20 cm at a 1:15 ratio, and is fully embedded in quartz sand. Experiments were conducted in a steel container (3.15 m × 1.1 m × 0.9 m) with sinusoidal excitation from 10 to 40 Hz (prototype: 0.94–1.89 Hz). For P-waves, the transmission ratio in the prototype band reaches a minimum of −30 dB (99.9% amplitude attenuation), and the P-wave response at 1.5 Hz is 99% lower than without the SMs. The innovation lies in integrating negative Poisson’s ratio structures into pile-type SMs, reducing the lower limit of the bandgap to 0.94 Hz. However, only P-waves were verified, and complex conditions like saturated soil were not simulated.
(2) **Suspended Mass-Type Periodic Metamaterials** [29]: Aluminum outer tube—polymer spring—steel inner core, as shown in Figure 1; scaled 1:30 (period 30 mm), 1 × 5/1 × 10/1 × 15 arrays in quartz sand. Prototype bandgap: 4–7 Hz (P/S), transmission min. −80 dB (99.99%) after 15 units, 90% energy attenuation under Northridge wave. Innovation: first optical rainbow trapping in SMs, 3× bandwidth vs. single-frequency units. Limitation: The modulus of polymer springs drifts by ±15% per year (temperature/humidity).
(3) **Three-Component Subwavelength Metamaterial Plates** [30]: This composite structure of “concrete-rubber-steel core” features a steel core as a high inertial body with a rubber layer regulating stiffness. The prototype period is 1.5 m, scaled down to 30 cm at a 1:5 ratio, and fully embedded in quartz sand. The plate thickness is 1/133 of the wavelength of a 5 Hz Rayleigh waves. Experiments in a glass container used sinusoidal excitation from 110 to 300 Hz (prototype: 0.5–9.0 Hz). For Rayleigh waves, the prototype exhibits an ultra-broadband bandgap of 0.5–9.0 Hz (bandwidth 8.5 Hz), with a 99% response reduction at 2.2 Hz. The bandgap width is four times wider than traditional two-component SMs. However, Love waves were not verified, and pore water in saturated soil may cause bandgap drift.
(4) **Foam-Steel Composite Plate Metamaterials** [31]: Steel block–foam plate unit; scaled 1:30 (lattice 5 cm), 1 × 2/1 × 4 arrays in quartz sand. Prototype bandgap: 3.5–10 Hz (Rayleigh), attenuation >70% after 4 rows. Innovation: bonding-only fabrication, 1/3 cost of lead-core rubber. Limitation: body waves unverified, foam aging shifts resonance.
(5) **Concrete Bed-Clamped Metamaterials** [32]: Cross-shaped steel resonator clamped to concrete bed (CEB), moment of inertia 1.8× circular piles; scaled 1:40 (bed thickness 12.5 mm), 5 × 3 array in sand. Prototype bandgap: 0–33 Hz (Rayleigh), 99% reduction at 5 Hz; bandgap stable in layered soil. Innovation: zero-frequency stopband covers full low-frequency range. Limitation: Love waves unverified; soil settlement may debond steel–concrete interface.
(6) **Petal-Shaped Metamaterials** [33]: Three-component unit of rubber–petal-shaped PMMA–steel; scaled 1:10 (lattice 10 cm), full embedment in sand. Prototype bandgap: 2.66–10 Hz (body/Lamb), peak acceleration reduced 94.2% under Oroville wave. Innovation: 2.66 Hz initial frequency overcomes low-frequency difficulty of traditional SMs. Limitation: 2D structure unsuitable for 3D waves; scaling effects cause bandgap deviation.
(7) **NPR Foam-Filled Pipe Metamaterials** [34]: Steel pipe filled with NPR foam (Poisson’s ratio −0.8); scaled 1:20 (pipe outer dia. 10 cm), square array, 0.5–1 m depth. Prototype bandgap: 0–16.34 Hz (Rayleigh), 99% reduction at 5 Hz; Rayleigh → body wave conversion at 45°. Innovation: bandgap width 2.7× that of traditional rubber filling. Limitation: saturated soil unverified; NPR foam stiffness drops when moist.
(8) **Buried Resonator Metabarrier** [35]: Aluminum shell, soft spring and steel mass resonators embedded in resin substrate, 12 rows of resonators arranged in triangular lattice with a scaled lattice constant of 1.7 cm, as shown in Figure 2; the scaled resonant frequency is 11.5 kHz. Prototype bandgap: 4.9–7.5 Hz, maximum surface motion attenuation up to 50% under Ricker wave excitation, and the attenuation effect increases with the number of resonator rows. Innovation: Realizes Rayleigh waves converting into shear bulk waves via local resonance, and bandgap formation is independent of Bragg scattering. Limitation: The resonator has a limited quality factor, and transient response effects lead to slight deviations between experimental and simulated results.
(9) **Nested Mass Metabarriers** [36]: Concrete core–PE foam–plywood shell; scaled 1:13.33 (lattice 30 cm), 8/16-unit arrays in quartz sand. Prototype bandgap: 0.8–3.2 Hz (P-wave), 78% attenuation under Taft wave (16 units). Innovation: initial frequency 0.8 Hz, suitable for ultra-low frequency. Limitation: multi-waveform coupling unverified, PE creep > 5%.
(10) **Rotational Oscillator-Type Metamaterial Foundations** [37]: Steel core–rubber–PMMA, core rotates about axis; scaled 1:25 (period 4 mm), 4 × 8 array, steel-cable suspension, connected to a four-story frame. Prototype bandgap: 2.2–11.4 Hz (plane/bending), top acceleration reduced 72% at 5 Hz. Innovation: bidirectional isolation, 90% bearing capacity vs. concrete foundations. Limitation: PMMA stiffness mismatch may cause deviation.
(11) **Local Resonance Metasurfaces for SH Waves in Granular Media** [38]: Forty-eight brass (10.2 g) + 3D-printed spring resonators, 4 × 12 array embedded 20 mm below glass bead surface in a 2000 × 1500 × 1000 mm box. Experimental band: 100–800 Hz. Periodic type: 330–400 Hz (prototype 5–8 Hz), attenuation −20 dB (90%); gradient type: 280–360 Hz (4–6.7 Hz), −25 dB (97%), propagation distance shortened 60%. Innovation: first subwavelength SH wave attenuation in granular media, gradient type broadens bandgap 30%. Limitation: only SH waves controlled, full-scale verification lacking.
(12) **Shallowly Buried Periodic Filled Pipe Barriers** [39]: Rubber pipe + filler, lattice constant 0.1 m, depth 0.1 m, square pattern, up to 20 × 5 columns in a 3.3 m × 2.3 m × 2.3 m concrete sand box. Experimental band: 139.2–178.1 Hz (prototype 53.6–85.8 Hz, subway-appropriate). Attenuation: average ARS 9.8 dB (89.7%) for N = 20; gradient layout broadens band to 30–80 Hz; 75% attenuation for Beijing Metro Line 13. Innovation: subwavelength shallow-buried structure, gradient layout, dual-mechanism integration. Limitation: deep burial unverified, relatively high frequency, complex soil and Love waves not considered.

Different from the low-frequency control relying on subwavelength units in the local resonance mechanism, Bragg scattering mainly achieves mid-to-high frequency regulation by matching the structural period with the seismic wavelength.

#### 2.1.2. Bragg Scattering-Dominated BSMs

Bragg scattering relies on a period ≈ λ/2 to create destructive interference and is suitable for mid-frequency waves (10–50 Hz), typically using periodic piles or trenches. Performance is summarized in Table 2.

**Figure 3:**
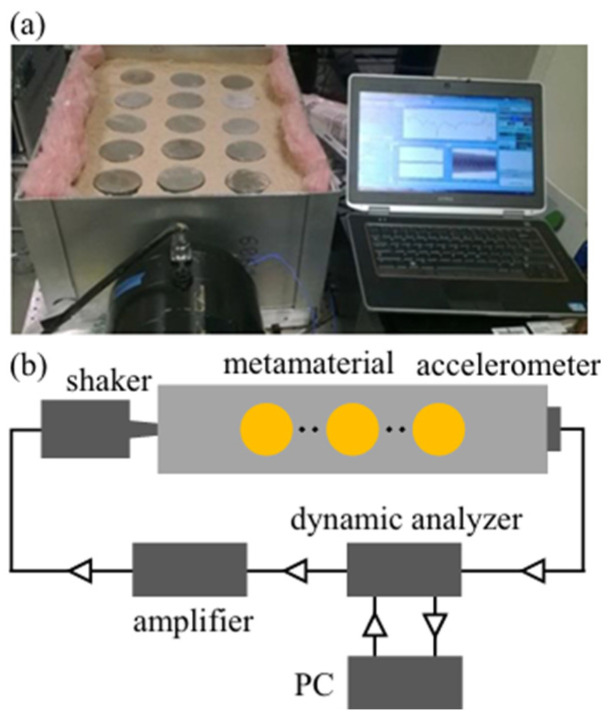
(**a**) Laboratory-scale experimental setup. (**b**) Schematic for the surface wave transmission testing.

(1) **Multi-Layer Soil Periodic Pile Metamaterials** [40]: Periodic concrete piles (solid/hollow) in multi-layer sand; scaled 1:10 (steel column dia. 7.7 cm), 3 × 5 array, as shown in Figure 3. For Rayleigh waves, shallow SMs (6 m depth) achieve 80% attenuation in 5–7 Hz; deep SMs (18 m depth) form 0–7.2 Hz cutoff bandgap; hollow piles maintain bandgap at 9.4% volume fraction. Innovation: utilizes natural soil layering, reduces initial frequency by 30%. Limitation: vertical P-waves unverified, soil liquefaction may alter pile–soil coupling stiffness.
(2) **Soil-Periodic Pile Systems** [41]: Hollow/filled steel pipe piles (outer dia. 20 cm, wall 1 cm) scaled 1:10, triangular lattice (period 40 cm) in silty clay. For P/S waves, filled piles show vertical bandgap 163–260 Hz (prototype 16.3–26 Hz), attenuation 98%; horizontal bandgap 230–375 Hz (prototype 23–37.5 Hz), 67 Hz wider than hollow piles. Innovation: quantifies filling effect on bandgap for engineering design. Limitation: hammer impact is instantaneous, no cyclic seismic simulation.

A single mechanism has inherent frequency band limitations; thus, multi-mechanism synergy and waveform conversion have become important research directions.

#### 2.1.3. Dual-Mechanism Synergistic and Waveform Conversion-Type SMs

This type integrates local resonance and Bragg scattering, and achieves broadband attenuation through waveform conversion (surface waves → body waves), covering broad low–mid frequencies. Key performance is summarized in Table 3.

**Figure 4:**
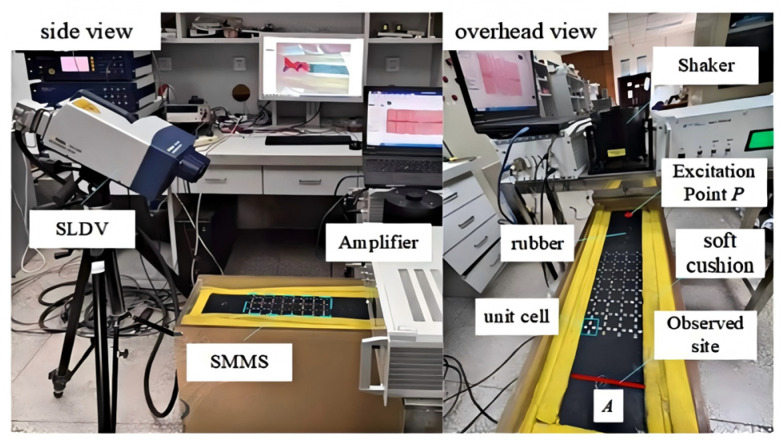
The lab-scale experiment setup for the surface wave transmission testing [44]. Reprinted under CC BY 4.0 license.

(1) **Periodic Capsule Barriers** [42]: PVC capsule–gas/concrete unit; scaled 1:10 (capsule dia. 24 cm), 3-row array in sand. GGC combination (2 gas + 1 concrete) forms XY-mode bandgap 391–452 Hz (prototype 3.9–4.5 Hz) and Z-mode bandgap 287–336 Hz (prototype 2.9–3.4 Hz), 68% attenuation under El Centro waves. Innovation: mixed filling broadens bandwidth by 30% vs. single filling. Limitation: PVC aging may cause gas leakage, affecting bandgap stability.
(2) **Cork Pile-Type Metamaterials** [43]: Concrete core–cork outer layer double-layer pile; scaled 1:5 (pile dia. 35 cm, cork thickness 10 cm), square array in quartz sand. For S-waves, hollow cork SMs show prototype bandgap 3.65–7.56 Hz, initial frequency 25% lower than solid piles; PGA reduced 75% under El Centro wave. Innovation: cork has negative carbon footprint, cost 50% lower than rubber SMs. Limitation: vertical P-waves unverified, cork modulus may increase when moist.
(3) **Rayleigh Wave Metasurfaces** [44]: Rectangular steel resonator–soil unit; scaled 1:50 (period 30 mm), 8 × 2 array in rubber blocks, as shown in Figure 4. Prototype bandgap: 3.2–19.2 Hz (Rayleigh), peak acceleration reduced 96.2% under Bishop seismic waves. Innovation: derives parameter design range, avoids trial-and-error. Limitation: complex soil not verified, heterogeneous soil may cause parameter deviation.
(4) **Gyroid-Type TPMS Metamaterials** [45]: Gyroid triply periodic minimal surface units; scaled 1:40 (lattice constant 5 cm), 3D-printed PLA, fully embedded in soil. For Rayleigh/P/S waves, prototype ultra-broadband bandgap 0–47.05 Hz, peak displacement reduced 75% under El Centro wave. Innovation: bandgap width 130% wider than traditional structures. Limitation: large-scale TPMS difficult to 3D print, requires segmented assembly.
(5) **Layered Rubber-Concrete Superstructures** [46]: Concrete plate–rubber layer–embedded resonator system; scaled 1:10 (plate 30 × 30 × 6 cm), 4 × 4 steel–rubber resonators, fully embedded in sand. For S-wave induced horizontal vibration, prototype bandgap 20–60 Hz, acceleration attenuation 77.8% at 30 Hz; the attenuation rate decreases by less than 3% after 100 cycles. Innovation: “load-bearing + vibration reduction” integration, concrete compressive strength 30 MPa. Limitation: vertical bandgap does not cover core 1–10 Hz range.
(6) **Seismic Metaconcrete Barriers** [35]: Concrete outer tube–elastic bearing–steel inner core; scaled 1:100 (tube dia. 2.2 mm), 12-line triangular array in resin blocks. For Rayleigh waves, prototype bandgap 4.9–7.5 Hz, displacement attenuation 90% after 32-line array; Rayleigh → body wave conversion dissipates 60 m underground. Innovation: mode conversion efficiency 70%, 133% higher than reflective SMs. Limitation: resin shear wave velocity much higher than actual soil, may cause bandgap offset.

### 2.2. Field Experiments

Field experiments of BSMs are the key link to verify engineering feasibility. Compared with laboratory scaled-down experiments, field experiments rely on real geological environments (layered soil, saturated soil), full-scale structures, and large vibration sources, directly connecting theoretical design with practical applications. This section classifies BSMs by physical mechanisms. Key performance is summarized in Table 4.

#### 2.2.1. Local Resonance-Dominated BSMs

In field experiments, this mechanism typically manifests as a “rigid mass block-flexible support” composite structure.

**Figure 5:**
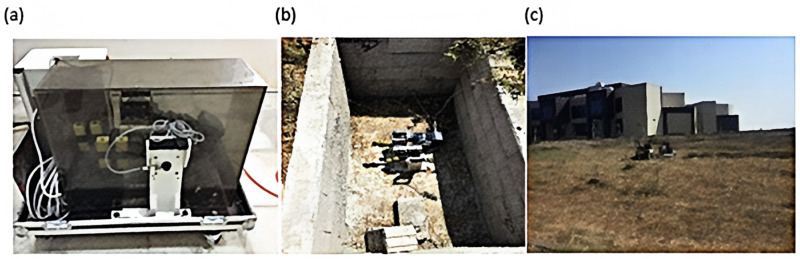
(**a**) Harmonic vibration source. (**b**) Location of harmonic source. (**c**) Experiment field [52]. Reprinted under CC BY 4.0 license.

(1) Local Resonator Array Barriers [47]: The core unit is a “TPU85A support-steel mass block-acrylic shell” (20 cm × 20 cm × 30 cm, steel block mass 10.53 kg). A 5 × 5 array of 25 units is fully embedded in a 4 m thick sand layer (16 m × 5 m × 5 m site). Sweep frequency excitation was applied from 10 to 100 Hz. For Rayleigh waves, the effective attenuation band is 20–100 Hz, with a minimum transmission ratio of −30 dB (99.9% attenuation) at 30 Hz. This is the first large-scale verification of the spacing regulation effect of local resonance units. However, only homogeneous sand was simulated, and horizontal vibration was not tested.
(2) **Metaconcrete Foundation Pit Backfill Barriers** [48]: Lead core–rubber coating–concrete matrix unit (lead core radius 0.12 m, rubber coat 0.12 m); multi-resonance type with 4 lead core sizes, barrier 1.2 m × 6.2 m, embedded as backfill. For Rayleigh waves, multi-resonance metaconcrete has FRF below −16 dB in 5–30 Hz; measured subway peak acceleration reduced 73.8%; single-resonance unit reaches −27 dB at 8.8 Hz, far exceeding traditional EPS. Innovation: first integration of SMs into foundation pit backfill. Limitation: mainly numerical simulation, lacks full-scale on-site verification.
(3) **Periodic Barriers of Rubber-Wrapped Concrete Piles** [49]: Concrete piles wrapped with rubber (pile length 1 m, inner dia. 0.06 m, outer dia. 0.08 m), square lattice (period 0.3 m), 2-row array in Langfang, Hebei site (four-layered soil). For Rayleigh/Love waves, attenuation bands 91.6–97.3 Hz (Rayleigh) and 93.9–99.4 Hz (Love); −28 dB (97%) at 95 Hz; at 30 km/h, Doppler effect broadens the bandgap to 40.4–50.8 Hz. Innovation: first simultaneous attenuation of Rayleigh and Love waves; layered soil design close to reality. Limitation: frequencies below 50 Hz unverified, soil compaction effect not evaluated.
(4) **Three-Component Composite Column Metasurfaces** [50]: Steel core–rubber coating–concrete rigid coating composite columns, square lattice embedded around Nanjing–Ningwu Railway. For Rayleigh waves, bandgaps 36.5–96.4 Hz (steel core) and 62.0–100 Hz (concrete core); −35 dB at 32 Hz; train vibration main frequency components attenuated >40%. Innovation: three-component structure broadens bandgap. Limitation: saturated soil not simulated, rubber aging may cause bandgap drift.
(5) **Periodic Composite Filled Trenches** [51]: EPS–concrete symmetrically filled trenches (period 0.4 m, width 0.2 m, depth 0.8 m), 8 rows along Dongguan Intercity Railway (miscellaneous fill–silty clay–granite residual soil). For Rayleigh waves, composite trench bandgap 48–65 Hz, average attenuation 96.78%, max −30 dB at 54 Hz; optimal at 0.8 m depth. Innovation: GPR detects layered soil parameters for accurate design. Limitation: body waves unverified, long-term EPS compression deformation affects performance.
(6) **Circularly Arranged Concrete Piles** [52]: Thirty-five concrete piles (dia. 15 cm, depth 2 m) in circular pattern (dia. 10 m), homogeneous soil at Iskenderun Technical University, Turkey, as shown in Figure 5. For Rayleigh waves, attenuation peaks at 5.1 Hz (−16 dB) and 14 Hz (−37.82 dB); displacement reduced 58% at 14 Hz. Innovation: circular array suitable for multi-directional incident waves. Limitation: Love waves unverified; soil settlement may cause pile inclination.
(7) **Modular Metamaterial Barriers** [53]: Concrete pyramid–stainless steel rod unit cells; 6 units in 3 × 2 × 1 array embedded in U-shaped concrete shell, depth 1 m at Ertz Railway Station, Germany. For Rayleigh waves, effective band 20–50 Hz, IL 10 dB at 31.5 Hz, 400% higher than empty shell. Innovation: modular design shortens construction period by 50%. Limitation: frequencies below 20 Hz unverified; steel rod corrosion affects long-term performance.
(8) **Triangularly Arranged Cylindrical Empty Holes** [54]: Three rows × 6 columns empty holes (dia. 0.3 m, depth 2 m), equilateral triangular pattern (period 1.5 m), Turkish sedimentary basin soil. For Love waves, bandgaps 4.8–6.7 Hz (80%) and 7.4–8.44 Hz (87%); peak displacement reduced 88% at 8 Hz. Innovation: subwavelength design breaks through low-frequency regulation. Limitation: body waves unverified; empty hole collapse risk.
(9) **Squarely Arranged Cylindrical Empty Holes** [55]: Empty holes (dia. 0.1143 m, depth 2 m), square pattern (period 1.5 m), Turkish sedimentary soil. For mixed Rayleigh–Love waves, bandgap 4.6–6.4 Hz, attenuation 80% at 5.5 Hz. Innovation: low-cost empty hole design. Limitation: narrow frequency band, fails to cover 1–4 Hz ultra-low range.

#### 2.2.2. Bragg Scattering-Dominated BSMs

In field experiments, this mechanism typically appears as periodic empty holes, pile arrays, or trenches.

**Figure 6:**
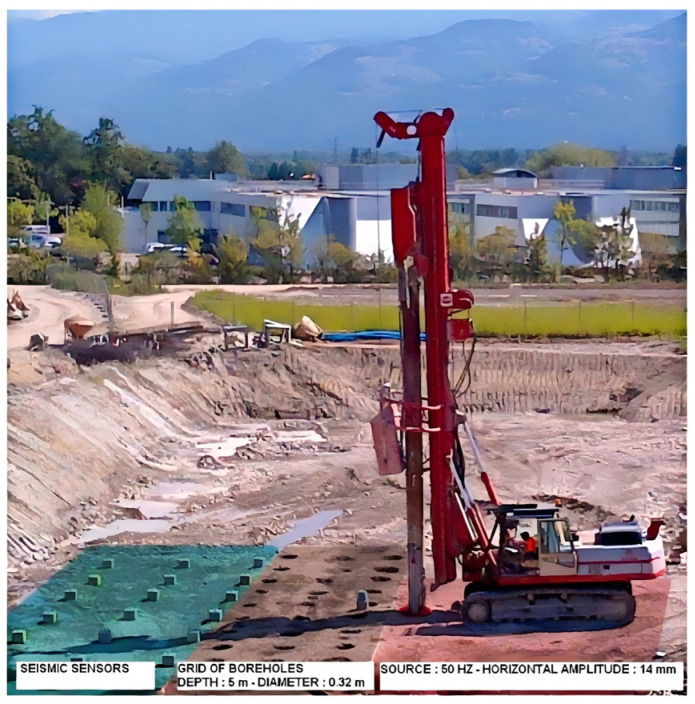
Experimental setup photograph. Sensors are distributed in the green region, and uniformly spaced vertical boreholes form the seismic metamaterial in the blue region. Reproduced from the supplemental material of Ref. [19]. Reprinted under CC BY 3.0 license.

(1) **Periodic Empty Hole Arrays (Bragg Scattering Paradigm)** [19]: Thirty-one empty holes (diameter 0.32 m, depth 5 m) arranged in a 3 × 10 grid (period 1.73 m) in French Grenoble silty clay. A vibrating probe operating at 50 Hz generates seismic waves within the red region, as shown in Figure 6. For Rayleigh waves, energy behind the SMs was reduced by 5 times at 50 Hz. This was the first full-scale verification of SMs. However, it was single-frequency (50 Hz), and the empty holes have no bearing capacity.
(2) **Sinusoidally Arranged Concrete Drilled Holes** [56]: Concrete-filled holes (diameter 0.15 m, depth 2 m) arranged in a y = sin (x) pattern with a period of 1 m, embedded in Turkish sandy clay. A harmonic vibration source (5–15 Hz) was used. For Rayleigh waves, the bandgap is 5.8–14.4 Hz, with an attenuation of −39 dB (99.99% energy blocking) at 10.2 Hz. The sinusoidal arrangement broadens the bandwidth by 40% compared to square arrangements. However, layered soil was not verified.
(3) **Two-Dimensional Periodic Foundations** [57]: Cast iron core–rubber–concrete units (period 0.254 m), homogeneous soil in Texas, USA, supporting a 1 m steel frame. For S-waves (bandgaps 40–42.5 Hz, 43–84.5 Hz) and P-waves (30–50 Hz, 52–100 Hz), S-wave attenuation reaches −66 dB at 46 Hz. Innovation: foundation–structure integration design. Limitation: low-frequency coverage below 30 Hz insufficient.
(4) **Trench-Type Periodic Barriers** [18]: Three-layer concrete–polyurethane–concrete units (thickness 0.28 m, depth 1.52 m), long/short barriers in Texas layered sandy soil. For Rayleigh/Love/P-waves, B2 (double short barriers) has bandgap 15–100 Hz (horizontal crossline), attenuation −30 dB; El Centro peak attenuation 92%. Innovation: multi-waveform omnidirectional regulation. Limitation: saturated soil unverified; polyurethane aging causes bandgap drift.
(5) **Barrier-Foundation Synergistic Systems** [58]: Periodic barriers + periodic foundations (five-layer composite), steel frame in Texas, USA. For multi-waveforms, bandgap 10–100 Hz; A2BL (periodic foundation + long barrier) achieves −35 dB in 32–39.7 Hz; active isolation 30% higher than passive. Innovation: source-end–foundation synergistic isolation. Limitation: reflection amplification exists in some bands (46–48 Hz).
(6) **Two-Dimensional Elastic Bandgap Crystals** [59]: Honeycomb/triangular lattice holes in marble (dia. 6 cm, depth 160 cm, period 14 cm), Spanish quarry. For Rayleigh waves (excited by steel ball impact, 0–40 kHz), honeycomb lattice has bandgaps 0–2 kHz and 7–15 kHz, attenuation 19 dB at 8 kHz. Innovation: pioneering full-scale experiment. Limitation: frequency band higher than core seismic range (1–5 Hz), period needs enlargement for engineering.

#### 2.2.3. Waveform Conversion and Negative Refraction-Type SMs

This type of SM relies on dynamic anisotropy of periodic structures to realize negative refraction and directional regulation of seismic surface waves. Such metamaterials are mostly implemented as periodic empty hole arrays in field tests, which can also produce bandgap attenuation effect for surface waves, and are applicable to regional protection scenarios.

**Figure 7:**
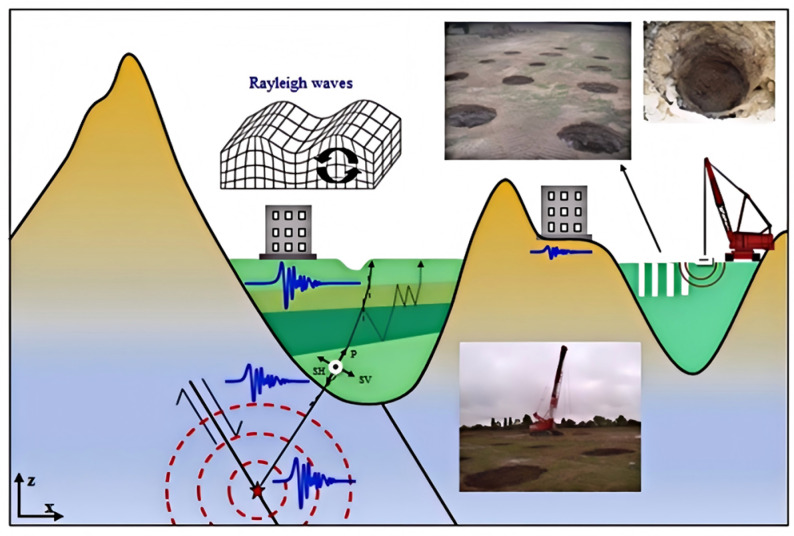
Schematic diagram of the field experimental setup for the periodic empty-hole seismic metamaterial flat lens, including the artificial seismic source, borehole array, and Rayleigh wave propagation [60]. Reprinted under CC BY 3.0 license.

(1) **Empty Hole Array Flat Lenses** [60]: Twenty-three empty holes (diameter 2 m, depth 5 m) arranged in a 45° inclined square pattern with a lattice period of 7 m, deployed in sedimentary strata near Lyon, as shown in Figure 7. Excited by a 17-ton drop hammer, the dominant frequency of generated Rayleigh waves is 8.15 Hz. Negative refraction occurs within 3–20 Hz (incident angle 15°, refraction angle −25°); the seismic response behind the array decreases by 88% at 8 Hz, and the energy focusing intensity reaches 2.3 times. Innovation: The first experimental verification of the flat lens effect for seismic waves. Limitation: The wave regulation performance is weak below 3 Hz, and the long-term stability of empty boreholes needs further monitoring.
(2) **Square Hole Array Metamaterials** [61]: A 6 × 7 periodic square hole array (lattice constant 1 m, hole side length 0.27 m, depth 0.35 m) constructed on stiff clay. This periodic hole structure also generates obvious bandgaps for surface waves: the bandgap ranges are 40–60 Hz for Rayleigh waves and 43–56 Hz for Love waves. A novel attenuation mode inversion phenomenon is found at 50 Hz: Love waves experience stronger attenuation within 40–50 Hz (−10 dB at 45 Hz), while Rayleigh waves show better attenuation within 50–60 Hz (−9 dB at 55 Hz). Innovation: The first discovery of bandgap-induced attenuation mode inversion of surface waves. Limitation: The attenuation characteristics below 40 Hz and the response to body waves remain unverified.

Overall, BSMs achieve the highest attenuation (up to 99.99%) and broad bandgaps but require deep excavation and high construction costs.

## 3. Experimental Research on Above-Surface Seismic Metamaterials (ASMs)

ASMs are placed directly on the ground surface, offering low cost, no excavation, and suitability for retrofitting existing structures. Taking local resonance as the dominant mechanism and accompanied by auxiliary Bragg scattering effects, they regulate seismic waves through periodic/resonant structures, with laboratory and field experiments focusing on surface wave attenuation (Rayleigh, Love, Lamb) and horizontal body waves (S-waves) below 60 Hz. Table 5 summarizes the core performance of ASMs.

### 3.1. Laboratory Experiments

Laboratory ASMs experiments (scales 1:10–1:100) validate ultra-low frequency isolation performance. Examples of key configurations, results, and limitations are detailed below.

**Figure 8:**
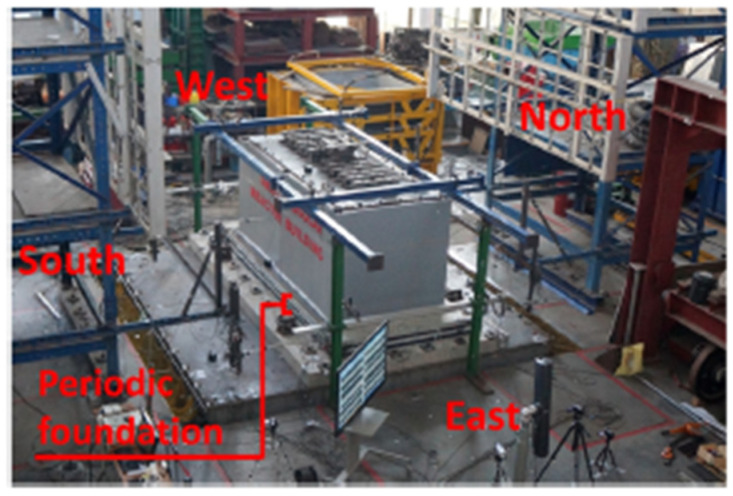
Full laboratory test setup of the 1:22 scaled 1D periodic foundation and small modular reactor model [65]. Reprinted under CC BY 4.0 license.

(1) **Curved boundary periodic metamaterials** [62]: Three-component rubber–PMMA–steel units with curved boundaries (in-plane stiffness reduced by 68%) at 1:30 scale, suspended in quartz sand. At 0–500 Hz excitation (prototype 0–16.7 Hz), Structure 2 produces three bandgaps (2.54–12.73 Hz total bandwidth, 2.4× Structure 1). TL reaches −40 dB (99% amplitude att.) at 10.9–16.7 Hz; Oroville PGA reduced by 98.13%. Innovation: curved boundary lowers initial frequency by 68%; multi-vibration modes broaden bandgap. Limitation: Love waves unverified; PMMA/rubber aging and full-scale validation lacking.
(2) **Tetra-chiral gradient metamaterials** [63]: Steel ligament–scatterer units at 1:50 scale, periodic/gradient layouts (rainbow trapping), fixed on sand surface with wires. Under 10–500 Hz sweep (prototype 0.2–10 Hz), periodic structure forms three bandgaps; gradient design broadens range to 2.5–30 Hz with TS = −25 dB at 8–14 Hz and Anza PGA reduced by 74%. Innovation: ultra-low 3 Hz bandgap; rainbow trapping broadens bandwidth to 27.5 Hz. Limitation: Love waves unverified; steel corrosion and ligament fatigue risk.
(3) **Sliding-resonance composite periodic foundations** [64]: Multi-layer concrete panels with embedded steel–silicone rubber resonators (SSR) at 1:5.5 scale, PTFE sliding layers for horizontal friction reduction. Under 0.5–9 Hz sinusoidal excitation (prototype 0.09–1.6 Hz), scaled bandgap 2.54–8.08 Hz (prototype 0.3–1.5 Hz); three-layer panels achieve 73% attenuation with El Centro PGA reduced by 75% (40% better than friction pendulum). Innovation: sliding-resonance composite covers ultra-low frequencies; modular design. Limitation: vertical seismic waves unisolated; silicone rubber/PTFE aging risk.
(4) **1D periodic foundation metamaterials** [65]: RC–polyurethane alternating layers at 1:22 scale for small modular reactor (SMR) isolation, as shown in Figure 8. Under white noise/seismic waves, bandgaps at 0.94–1.09 Hz (horizontal/torsional) and 1.48–1.64 Hz (vertical); SMR roof PGA reduced by 91.55% (Bishop). Innovation: single-unit 1D structure achieves omnidirectional isolation; bandgap load correction suits heavy nuclear facilities. Limitation: vertical 1–10 Hz bandgap missing; full-scale RC/polyurethane durability unverified.
(5) **Granular layer surface resonator metamaterials** [66]: Steel resonator–silicone bead layer with metal wire root fixation at 1:100 scale, 12 units linearly arranged. Under 2 kHz Ricker pulses (prototype 20 Hz), heavy resonator achieves TS = −12 dB (75% att.) at 700 Hz (prototype 7 Hz); propagation range reduced by 75%. Innovation: particle consolidation solves heterogeneous media impedance matching; dual-mode resonance covers two guided wave orders. Limitation: large scaling deviation; body waves unverified; wire root corrosion risk.
(6) **Composite foundation SMs** [67]: Concrete plates with embedded steel–rubber resonators, steel–teflon ultra-low damping interfaces. Under 0.5–8 Hz sweep (prototype 4.5–80 Hz), bandgap 4.5–8 Hz with 50% displacement reduction at 6 Hz; bearing capacity 50% higher than rubber bearings. Innovation: periodic system bandgap + dual-stiffness structure integration. Limitation: longitudinal wave att. <30%; 14 Hz high-frequency band unverified; rubber aging risk.

Following laboratory validation, field experiments further examine the vibration attenuation performance of ASMs in real surface environments.

### 3.2. Field Experiments

ASMs field experiments rely on real surface environments (soil, vegetation coverage), full-scale/large-scale structures, and natural/artificial excitations. These SMs target surface waves (Rayleigh, Love) and horizontal body waves (S-waves), achieving broadband attenuation below 60 Hz through structural innovation (inverted T-shape) or natural ecological structures (forests) [68]. This section classifies experiments into “artificially designed” and “natural ecological” types.

#### 3.2.1. Artificially Designed SMs

**Figure 9:**
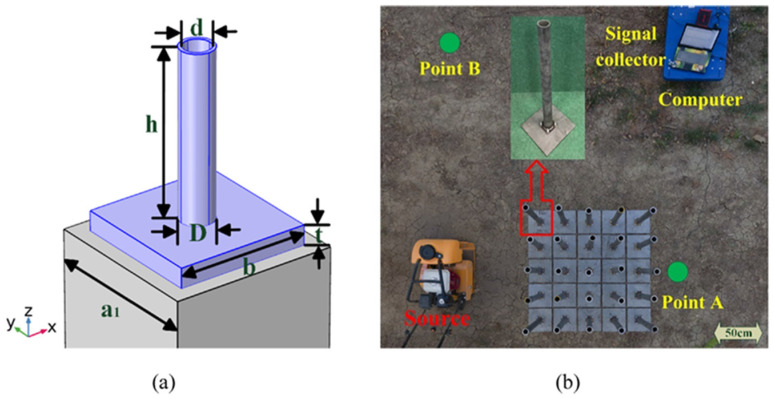
(**a**) Schematic of the unit cell; (**b**) top view of the experimental setup. Points A and B are signal acquisition points, and point B is the reference point [69]. Reprinted under CC BY-NC-ND 4.0 license.

(1) **Inverted T-shaped periodic metamaterials** [69]: Steel column–flat plate units in 5 × 5 square array at Tianjin University site (5 m × 5 m) (see Figure 9). Rayleigh waves from 20 kN hammer excitation; 1D bandgap 6.7–17.2 Hz (relative bandwidth 0.88), 2D array verified 34–130 Hz with −60 dB (99.9999% att.) at 44 Hz. Innovation: relative bandwidth 1.17, 134% higher than traditional columnar SMs; cost 1/3 of traditional bearings. Limitation: Love/body waves unverified; soil settlement may cause inclination.
(2) **Rubber column resonance metasurfaces** [70]: Rubber columns on coarse sand granular layer (lattice constant 0.5 m), eccentric vibrator 20–70 Hz. Love waves attenuation 5–8 dB better than Rayleigh; core bandgap 25–39 Hz; central sensor energy −20 dB (90%) at 28 Hz. Innovation: first confirmation of preferential Love waves attenuation by surface SMs; no excavation, cost 1/3 of traditional isolation. Limitation: body waves unverified; rubber aging affects long-term stability.

#### 3.2.2. Natural SMs

**Figure 10:**
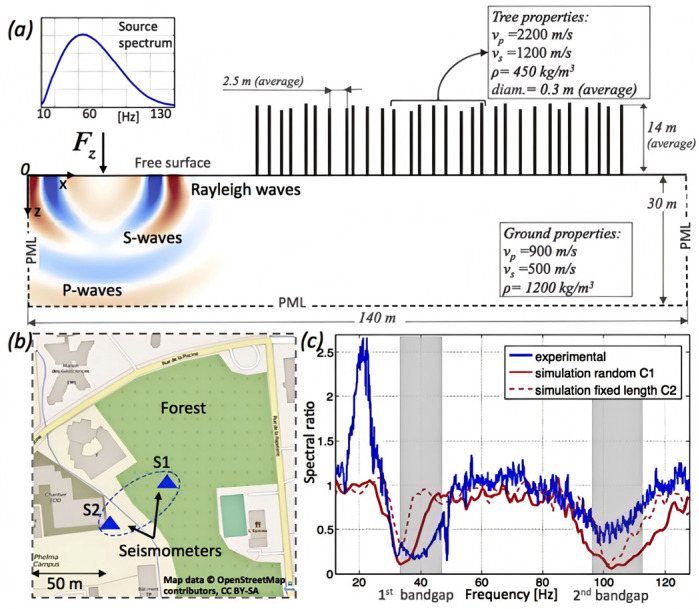
(**a**) The 2D computational domain. (**b**) Map of the forest location where S1 and S2 are the seismometers. (**c**) Measured (blue) and simulated (red) spectral ratios [72]. Reprinted under CC BY 4.0 license.

(1) **Landes forest natural metamaterials** [71]: A 120 m × 120 m pine forest (DBH 0.3 m, height 10 m, period ≈ 4 m), 961 vertical + 100 three-component geophones, 70 kg source 10–100 Hz. Bandgap 40–60 Hz with −25 dB (90% att.) at 45 Hz; Rayleigh wave attenuation length reduced from >2 λ to λ; energy blocking 93–99%. Innovation: first geophysical-scale natural SMs confirmation; zero cost, eco-friendly. Limitation: bandgap uncontrollable; tree lodging/aging destroys periodicity.
(2) **Grenoble pine forest natural metamaterials** [72]: A 60,000 m^2^ random array (height 10–20 m, DBH 0.2–0.4 m, spacing 1.5–4 m), ambient noise monitoring (see Figure 10). Dual bandgaps at 30–45 Hz (first-order) and 90–110 Hz (third-order); spectral ratio 0.17 (83% att.) at 37 Hz; propagation range reduced 80%. Innovation: natural dual bandgaps from tree multi-order resonance; low-cost ambient noise method. Limitation: single waveform coverage; bandgap affected by tree growth.

ASMs are low-cost and excavation-free but limited to higher frequencies and lower attenuation.

## 4. Experimental Research on Partially Embedded Seismic Metamaterials (PESMs)

PESMs combine underground fixation with above-ground resonance, balancing wave coupling efficiency and structural stability. They are suitable for seismic waves regulation in shallow geological conditions (sand, clay). Table 6 shows the characteristics of PESMs.

### 4.1. Laboratory Experiments

A series of scaled-down laboratory experiments further examined PESM performance under controlled conditions. Examples of key configurations, results, and limitations are detailed below.

**Figure 11:**
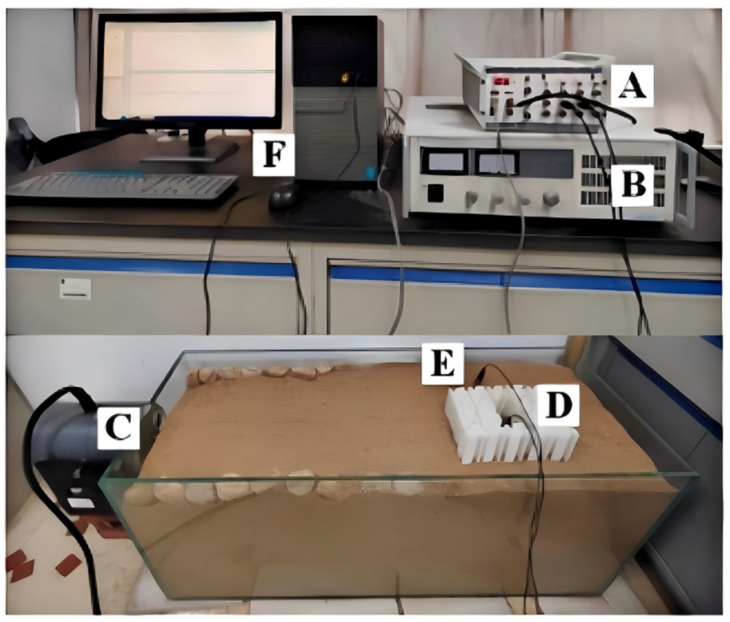
Experimental setup of partially embedded scaled gammadion-shaped chiral seismic metamaterials. A: Signal generator; B: Power amplifier; C: Vibration exciter; D: Metamaterial sample; E: Reference accelerometer; F: Computer [76]. Reprinted with permission under CC BY 4.0 license.

(1) **Negative Poisson’s Ratio Foam-Filled Concrete Square Column Metamaterials** [73]: Concrete square columns with NPR foam at 1:10 scale, 1/3 burial depth, 1 × 5 array in quartz sand. Under 20–230 Hz excitation (prototype 2–23 Hz), experimental bands 38–52 Hz (prototype 3.8–5.2 Hz, −30 dB/99.9%) and 70–186 Hz (7–18.6 Hz); Bishop PGA reduced 80.9%. Innovation: first NPR foam use in partially embedded SMs; ultra-broadband starting from 0 Hz. Limitation: only five units cause data deviation; foam aging; Love/body waves unverified.
(2) **Inertial Amplification-Local Resonance Coupled Metamaterials** [74]: Steel columns with inclined epoxy connectors at 1:50 scale, 4 × 4 array in saturated sand (burial 10 mm, exposure 20 mm). Prototype bandgap 5.3–13 Hz (scaled 300–550 Hz); T = −20 dB (99%) at 12 Hz; El Centro x/y acceleration reduced 74%/85%. Innovation: dual mechanism broadens bandwidth 194% vs. single LR. Limitation: only saturated sand verified; epoxy connector prone to settlement failure; Love waves unverified.
(3) **Split-Type Pseudo-Surface Wave Attenuating Metamaterials** [75]: Four fan-shaped concrete columns with cross-shaped above-ground structure (no rigid connection) at 1:100 scale, 5 × 6 array in saturated sand. Prototype bandgap 1.58–14.55 Hz (relative bandwidth 8.5); T = −54 dB (99.997%) at 12 Hz; Taft acceleration reduced 79%. Innovation: first pseudo-surface wave attenuation; relative bandwidth 8.5. Limitation: body waves unverified; polystyrene aging; single soil type.
(4) Gammadion-Shaped Chiral Seismic Metamaterials [76]: Partially embedded gammadion-shaped chiral pillars (epoxy scaled specimens) at 1:100 scale, 4 × 5 array arranged in sand, as shown in Figure 11. Prototype attenuation band 2.3–18 Hz; peak attenuation reaches 76.45% under amplitude-modulated seismic waves. Innovation: Chiral structure produces torsional–translational coupling, relative bandgap width up to 77.34% accompanied by negative effective mass density; good robustness to different burial depths. Limitation: Love waves and body waves unverified; medium inhomogeneity and interface slippage cause simulation-experiment deviation; only single sand medium adopted.
(5) **Subwavelength Rainbow Trapping Metamaterials** [77]: Polymer columns with steel mass blocks at 1:400 scale, 15-unit gradient array in clay (burial 1.5 mm). Prototype bandgap 7.5–9.85 Hz (relative bandwidth 0.32); η = −40 dB (99.99%) at 7.5 Hz; Northridge PGA reduced 75%. Innovation: subwavelength dual-mechanism synergy; prototype depth only 0.6 m. Limitation: only clay verified; polymer–clay interface failure risk; Love waves unverified.
(6) **Granular Medium Surface Resonance Metamaterials** [78]: Total of 100 3D-printed resonators in glass beads at 1:100 scale, 20 × 5 array (burial 24 mm). At 410 Hz (prototype 4.1 Hz), −20 dB (90%) att. in 200–650 Hz; propagation range reduced from 850 mm to 200 mm. Innovation: stiffness gradient achieves high-order mode down-conversion; shallow burial suits loose soil. Limitation: scaling deviation; actual soil unverified; particle consolidation affects depth.
(7) **Multi-Layer Resonance Granular Medium Metabarriers** [79]: One-to-three-layer resonator arrays in glass beads at 1:100 scale, 4 × 8 units per layer. At 410–500 Hz (prototype 4.1–5 Hz), two-layer bandwidth 33% wider than one-layer; three-layer guided wave range reduced 80%. Innovation: two-layer optimal design; stiffness gradient enhances coupling. Limitation: particle nonlinearity under strong earthquakes unsimulated; ABS shell needs replacement.

### 4.2. Field Experiments

Field experiments were conducted to validate PESMs performance at full scale. Examples of key configurations, results, and limitations are detailed below.

**Figure 12:**
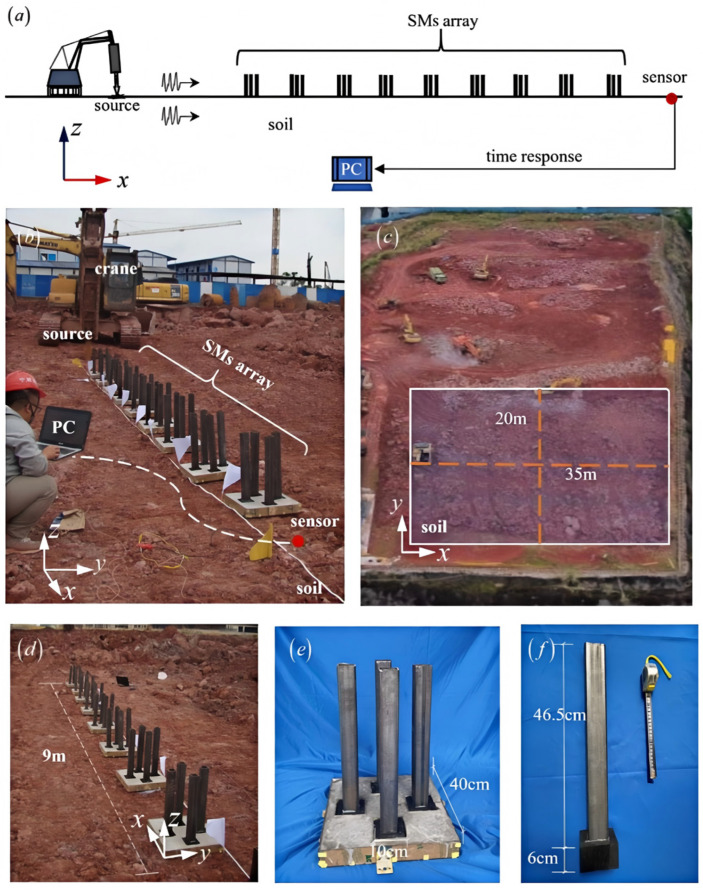
Experimental setup and metamaterial design: (**a**) schematic diagram; (**b**) field setup; (**c**) top view of the experimental area; (**d**) metamaterial array; (**e**) resonant unit; (**f**) rubber–steel connection [80]. Reprinted under CC BY-NC-ND 4.0 license.

(1) **Collective Eigenmode-Driven Subwavelength Metamaterials** [80]: Composite unit of underground concrete platform with above-ground rubber-steel pipe; partially embedded, 1 × 9 linear array in homogeneous soil in Chongqing (see Figure 12). For Rayleigh waves, numerical bandgap 9.18–9.32 Hz; field band 10.1–10.78 Hz; acceleration reduced 88% at 10 Hz; propagation range reduced from 20 × 35 m^2^ to 5 m. Innovation: first use of collective eigenmodes for subwavelength bandgaps; mixed arrangement increases resonance efficiency by 40%; cost is 1/5 of traditional seismic isolation. Limitation: narrow bandgap; suitable only for homogeneous soil; long-term stability unverified.
(2) **Full-Scale 2D Semi-Embedded Rubber Column Metamaterials (SEM)** [81]: Rubber columns (0.1 m × 0.1 m × 0.4 m) in 6 × 7 periodic array, semi-buried in 5.0 m × 4.0 m × 0.5 m sandy substrate. For Love and Rayleigh waves (20–70 Hz excitation), global bandgap 25–37 Hz; local bandgap 37–42 Hz; Love wave attenuation −9.3 dB at 31 Hz, superior to Rayleigh (−5.3 dB). Mechanism: exposed column resonance dominates energy dissipation; underground section introduces damping that slightly reduces efficiency. Innovation: confirms SEM protection potential for slender structures (bridges, skyscrapers); provides “prioritize damping characteristic regulation” criterion for flexible resonator design. Limitation: Love–Rayleigh attenuation gap; rubber aging risk.

PESMs balance performance and constructability, ideal for shallow soils but sensitive to soil conditions.

## 5. Experimental Research on Generalized SMs

Generalized SMs achieve seismic protection indirectly by optimizing structural response (suppressing bending vibration) and improving impact energy absorption, rather than directly regulating seismic waves. Table 7 summarizes core performance. Key configurations, results, and engineering potential are detailed below.

**Figure 13:**
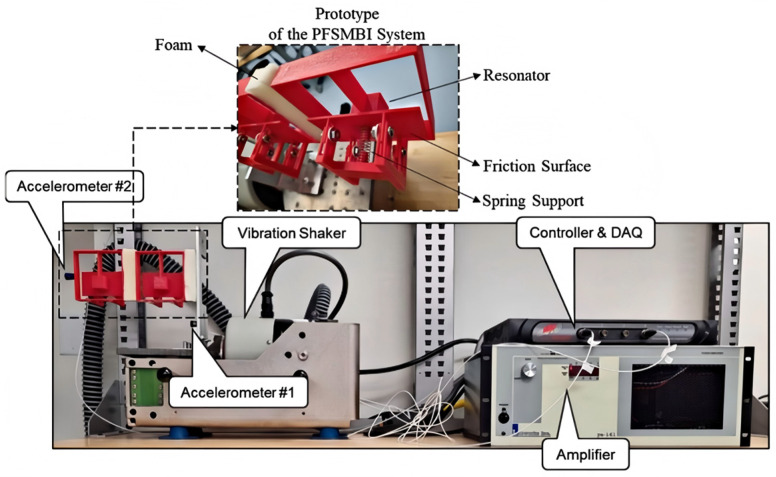
Experimental setup for dynamic testing of the PFSMBI prototype under harmonic excitation [83]. Reprinted under CC BY 4.0 license.

(1) **Cantilever-Type Local Resonance Metamaterials** [82]: All-metal cantilever units (8–32 units, lattice constant 450 mm) arranged on H-shaped steel beam web, tested under PCB impact hammer with 10–50 Hz white noise excitation, measured by three acceleration sensor groups. For horizontal bending vibration (corresponding to Rayleigh and S-wave responses), 32-unit full-scale bandgap is 17.49–18.13 Hz (FRF = −18 dB, 98% attenuation); bandgap reduces to 0.92 Hz (covering 0–10 Hz) after gypsum material + 4× geometric amplification; displacement attenuation in 0.92–1.8 Hz band under El Centro waves reaches 73%. Innovation: all-metal structure solves soft material aging; “material + geometric scaling” covers wide frequency band; modular installation is fast. Limitation: vertical vibration uncontrolled; fatigue/corrosion tests lacking; no on-site building verification.
(2) **Passive Friction Seismic Metamaterial Base Isolator (PFSMBI)** [83]: Periodic lattice units (1–4 cells in four layout forms, cell dimension 150 cm) connected by viscoelastic layers, tested via harmonic excitation and three actual seismic wave records (see Figure 13), verified by 2-cell 3D-printed small-scale prototype and finite element simulation within 0–20 Hz low-frequency range. The proposed isolator forms low-frequency bandgaps; the optimized unit cell achieves bandgap of 10.942–21.718 Hz, effectively reducing floor acceleration and inter-story drift of six-story buildings. Coulomb friction model overestimates energy dissipation, while LuGre model accurately reflects stick–slip motion (SSM); under seismic excitation, friction energy dissipation ratio declines notably when SSM occurs, and locked resonators cause response amplification. Innovation: Friction-based solid energy dissipation avoids liquid leakage issues; integrated local resonance and bandgap characteristics realize low-frequency seismic isolation; multiple cell layouts and adjustable parameters enable flexible performance optimization.
  Limitation: Excessive friction triggers stick–slip motion and degrades damping effect; only small-scale prototype tests conducted; no long-term durability and field engineering application research.
(3) **Porous Auxetic Thin-Walled Cylindrical Tubes** [9]: Auxetic grid (diamond, square, peanut-shaped CPG) + thin-walled tube laser-cut from ST-37 steel (outer diameter 60 mm, wall thickness 2 mm, length 110 mm); tested under SANTAM universal testing machine (quasi-static compression at 5 mm/min, simulating P-wave impact). For axial impact (corresponding to P-waves), CPG-3.5 tube (porosity 31.8%) achieves specific energy absorption (SEA) of 7.18 J/g (139% higher than traditional steel tubes); impact absorption efficiency for magnitude 6–8 earthquakes reaches 78%; SEA attenuation <10% after three cycles. Innovation: CPG grid optimizes stress distribution; porosity adjustable; cost 40% lower than 3D printing. Limitation: SEA decreases 20–30% under multi-directional loads; dynamic tests lacking; no large-scale verification.
(4) **Dual-Helix Inertial Amplification Metamaterial Beams (HMBI)** [84]: Aluminum alloy integrated unit cell (3 turns antisymmetric double helix) with steel mass ball at center, 15 units forming metamaterial beam; tested under Thorlabs piezoelectric stack (0–2000 Hz sinusoidal sweep), mid-span displacement measured by laser vibrometer. For vertical bending vibration (corresponding to Rayleigh and S-wave responses), HMB bandgap 430–670 Hz (T = −30 dB, 95% attenuation); HMBI inertial amplification reduces bandgap to 301–529 Hz (T = −25 dB); 10× unit amplification further reduces to 30–53 Hz (covering 0–50 Hz); displacement attenuation under El Centro waves in 30–53 Hz band reaches 78%. Innovation: double helix reduces initial bandgap frequency by 40%; inertial amplification enables quantitative frequency regulation. Limitation: only vertical bending vibration controlled; accelerated aging and dynamic fatigue tests lacking; no large-scale prototype verification.

## 6. Summary, Comparison, and Future Outlook

Research on SMs has advanced from theoretical exploration and laboratory validation toward practical engineering translation, with BSMs, ASMs, and PESMs emerging as the three primary technical pathways. This section synthesizes common research advances, systematically compares their performance and applicability, identifies key unresolved challenges, and outlines a phased roadmap for future deployment.

### 6.1. Research Progress: Common Breakthroughs and Differentiated Characteristics

#### 6.1.1. Common Progress

Across BSMs, ASMs, and PESMs, three representative advances have been widely corroborated through experiments:

(1) **Systematic verification of bandgap performance**: Both laboratory-scale and field experiments demonstrate that SMs generally achieve attenuation ratios exceeding 70% within the critical seismic frequency range of 0–50 Hz. BSMs exhibit maximum attenuation up to 99.99%, ASMs (e.g., forest-inspired configurations) attain 93–99% reduction of Rayleigh waves, and PESMs present bandgaps concentrated in the 1.5–14.5 Hz range, closely aligning with building-sensitive frequencies.
(2) **Mechanistic integration and bandwidth enhancement**: SM designs have evolved from single local resonance or Bragg scattering mechanisms toward synergistic hybrid strategies, including resonance–scattering coupling and surface-to-body wave conversion, which significantly broaden effective attenuation bandwidth [23].
(3) **Full-scale validation of topological characteristics**: Recent field experiments have confirmed topological interface effects in SMs, providing enhanced robustness against soil heterogeneity and structural imperfections [26]. Additionally, advanced structural optimization under nonstationary stochastic seismic loading has further refined the dynamic modeling and design framework for SMs, supporting more realistic performance assessment [27].

It is worth pointing out that most results stem from laboratory-scale trials and field investigations, and related information on uncertainty and repeatability remains underreported.

#### 6.1.2. Differentiated Characteristics

Despite shared attenuation mechanisms, BSMs, ASMs, and PESMs exhibit distinct performance features and engineering applicability, as summarized in Table 8.

BSMs deliver the most robust attenuation and stable performance but require deep excavation and substantial construction investment, rendering them more suitable for newly built critical infrastructure. ASMs eliminate excavation requirements and offer economic advantages, making them ideal for retrofitting existing structures, albeit with relatively higher effective frequencies. PESMs achieve a favorable balance between attenuation efficiency and construction feasibility, adapting well to shallow soil conditions with moderate seismic demands.

### 6.2. Existing Limitations

Four core challenges persist in translating SM technology into reliable engineering practice:

(1) **Scaling-induced performance deviations**: Laboratory experiments typically employ idealized materials and boundary conditions, while full-scale applications encounter complex soil nonlinearity and stiffness variations, leading to bandgap shifts and attenuation degradation.
(2) **Inadequate adaptability to complex geology**: Most experimental investigations assume homogeneous media, whereas layered, saturated, or frozen soils substantially alter wave propagation characteristics and SM–soil interaction efficiency.
(3) **Insufficient long-term durability**: Aging effects, environmental corrosion, and material degradation progressively disrupt the stiffness matching between resonators and host media, compromising long-term performance stability.
(4) **Incomplete multi-waveform regulation**: Current studies predominantly focus on Rayleigh wave attenuation, while Love wave and body wave mitigation remain insufficiently explored, limiting performance under multi-component seismic excitation.

### 6.3. Future Outlook

To address the above limitations and facilitate systematic engineering deployment, four priority research directions are proposed:

(1) **Multi-waveform collaborative attenuation**: Develop integrated SM configurations capable of simultaneous suppression of Rayleigh, Love, and body waves, achieving comprehensive coverage of the 0–10 Hz building-sensitive frequency band.
(2) **Soil–SMs dynamic matching**: Establish quantitative design frameworks linking soil stratigraphy, groundwater conditions, and SM parameters, enabling adaptive modular designs for variable geological environments.
(3) **Durability-oriented optimization**: Adopt corrosion-resistant materials and modular replaceable components, ensuring stable performance over a 30-year design lifespan under realistic environmental conditions.
(4) **Full-scale demonstration and intelligent adaptation**: Conduct systematic field validations under representative site conditions and integrate smart materials to enable real-time bandgap tuning under time-varying seismic inputs.

A phased roadmap is proposed for SMs engineering implementation, aligned with current technical maturity and practical constraints:

- ASMs: 3–5 years (low cost, rapid deployment for retrofits);
- PESMs: 5–7 years (balanced performance, suitability for shallow soil regions);
- BSMs: 7–10 years (ultra-high attenuation, deployment for new critical infrastructure).

### 6.4. Summary

SM research has transitioned from conceptual innovation to application-oriented development, with BSMs, ASMs, and PESMs forming complementary technical systems. Bridging the gap between laboratory findings and engineering reliability requires concerted efforts to mitigate scaling effects, enhance geological adaptability, improve long-term durability, and expand multi-waveform regulation capability. Targeted deployment strategies should be adopted for different scenarios: ASMs for retrofitting, PESMs for shallow soil regions, and BSMs for new critical infrastructure. Addressing these challenges will enable SMs to become a viable and robust solution for seismic hazard mitigation in future engineering practice.

## Figures and Tables

**Table 1:** Performance of local resonance-dominated BSMs in laboratory tests.

Structure Type	Core Unit	Scale Ratio	Effective Frequency (Prototype)	Max Attenuation	Key Limitations
Auxetic pile	Concrete–TPU auxetic layer	1:15	0.94–1.89 Hz (P-wave)	99.9%	Only P-wave verified
Three-component plate	Concrete–rubber–steel	1:5	0.5–9.0 Hz (Rayleigh)	99%	Saturated soil ignored
Foam–steel plate	Steel–foam	1:30	3.5–10 Hz (Rayleigh)	>70%	Foam aging risk
Suspended mass	Aluminum–polymer spring–steel	1:30	4–7 Hz (P/S)	99.99%	Modulus drift ±15%/year
Clamped concrete	Cross steel–concrete base	1:40	0–33 Hz (Rayleigh)	99%	Debonding in soft soil
Petal-shaped	Rubber–PMMA–steel	1:10	2.66–10 Hz (body)	94.2%	Poor 3D wave adaptability
NPR foam pipe	Steel–NPR foam	1:20	0–16.34 Hz (Rayleigh)	99%	Stiffness drop when wet
Nested mass	Concrete–PE foam–plywood	1:13.33	0.8–3.2 Hz (P)	78%	PE creep > 5%
Rotational oscillator	Steel–rubber–PMMA	1:25	2.2–11.4 Hz (plane)	72%	PMMA stiffness mismatch
SH metasurface	Brass–3D printed spring	—	5–8 Hz (SH)	97%	Only SH wave tested
Shallow filled pipe	Rubber pipe–filler	—	53.6–85.8 Hz (Rayleigh)	89.7%	High frequency, complex soil unsuitable

**Table 2:** Performance of Bragg scattering-dominated BSMs in laboratory tests.

Structure Type	Core Unit	Scale Ratio	Effective Frequency (Prototype)	Max Attenuation	Key Limitations
Multi-layer periodic pile	Solid/hollow concrete pile	1:10	5–7 Hz (shallow); 0–7.2 Hz (deep)	80%	Vertical P-waves not verified; soil liquefaction risk
Soil–periodic pile system	Hollow/filled steel pipe pile	1:10	16.3–26 Hz (vertical); 23–37.5 Hz (horizontal)	98%	Instant hammer load; no cyclic seismic simulation

**Table 3:** Performance of dual-mechanism/waveform conversion BSMs in laboratory tests.

Structure Type	Core Unit	Scale Ratio	Effective Frequency (Prototype)	Max Attenuation	Key Limitations
Rayleigh wave metasurface	Rectangular steel resonator–soil	1:50	3.2–19.2 Hz	96.2%	Complex soil conditions ignored
Periodic capsule barrier	PVC capsule–gas/concrete	1:10	3.9–4.5 Hz (XY); 2.9–3.4 Hz (Z)	68%	PVC aging and gas leakage
Cork pile metamaterial	Concrete core–cork layer	1:5	3.65–7.56 Hz (S-wave)	75%	Vertical P-waves not verified
Gyroid-type TPMS metamaterial	Gyroid lattice	1:40	0–47.05 Hz	75%	Large-scale fabrication difficulty
Layered rubber–concrete superstructure	Concrete plate–rubber–resonator	1:10	20–60 Hz	77.8%	1–10 Hz frequency gap
Seismic metabarrier	Concrete tube–elastic bearing–steel core	1:100	4.9–7.5 Hz	90%	Resin–soil stiffness mismatch

**Table 4:** Key performance of BSMs in field experiments.

Mechanism	Structure Type	Effective Frequency Band	Maximum Attenuation	Site/Application
Local resonance	Local resonator array barrier	20–100 Hz	99.9%	Large-scale test site
Local resonance	Rubber-wrapped concrete pile	91.6–97.3 Hz	97%	Langfang, China
Bragg scattering	Sinusoidal concrete borehole	5.8–14.4 Hz	99.99%	Turkey sedimentary soil
Waveform conversion	Empty-hole flat lens	3–20 Hz	88%	Lyon, France

**Table 5:** Key performance of ASMs in experimental studies.

Structure Type	Core Unit	Type	Effective Frequency (Prototype)	Max Attenuation	Key Limitations
Curved boundary periodic	Rubber–PMMA–steel block	Lab	2.54–12.73 Hz (Lamb/P/S)	TL −40 dB (99%) at 10.9–16.7 Hz; PGA −98.13% (Oroville)	Love waves unverified; PMMA/rubber aging not evaluated
Tetra-chiral gradient	Steel ligaments–scatterers	Lab	2.5–30 Hz (Rayleigh)	TS −25 dB at 8–14 Hz; PGA -74% (Anza)	Love waves unverified; steel corrosion risk
Sliding-resonance composite foundation	Concrete + steel-silicone resonator (SSR)	Lab	0.3–1.5 Hz (S-waves)	73% att. at 0.3–1.5 Hz; PGA −75% (El Centro)	Vertical waves unisolated; PTFE/silicone aging risk
Granular layer surface resonator	Steel resonator + silicone bead layer	Lab	4–15 Hz (guided waves)	TS −12 dB (75% att.) at 700 Hz	Body waves unverified; wire root corrosion risk
1D periodic foundation	RC–polyurethane alternating layers	Lab	0.94–1.09 Hz, 1.48–1.64 Hz (S/P/torsional)	SMR roof PGA −91.55% (Bishop)	Vertical 1–10 Hz bandgap missing; polyurethane durability unverified
Composite foundation	Concrete plate + embedded resonators	Lab	4.5–8 Hz (S-waves)	Displacement −50% at 6 Hz; bearing capacity + 50%	Longitudinal wave att. < 30%; rubber aging risk
Inverted T-shaped periodic	Steel column + flat plate	Field	34–130 Hz (Rayleigh)	−60 dB (99.9999%) at 44 Hz	Love/body waves unverified; soil settlement risk
Rubber column resonance	Rubber columns on granular layer	Field	25–39 Hz (Love/Rayleigh)	Energy −20 dB (90%) at 28 Hz	Body waves unverified; rubber aging risk
Landes forest (natural)	Pine trees (DBH 0.3 m, height 10 m)	Field	40–60 Hz (Rayleigh)	−25 dB (90%) at 45 Hz; energy blocking 93–99%	Bandgap uncontrollable; tree lodging/aging
Grenoble pine forest (natural)	Random pine array (height 10–20 m)	Field	30–45 Hz, 90–110 Hz (Rayleigh)	Spectral ratio 0.17 (83%) at 37 Hz; range reduction 80%	Single waveform; bandgap affected by tree growth

**Table 6:** Key performance of PESMs in experimental studies.

Structure Type	Core Unit	Type	Effective Frequency (Prototype)	Max Attenuation	Key Limitations
Negative Poisson’s ratio foam-filled concrete column	Concrete column + foam	Lab	3.8–5.2 Hz; 7–18.6 Hz (surface)	−30 dB (99.9%) at 38–52 Hz; Bishop PGA −80.9%	Only 5 units; foam aging; Love/body waves unverified
Inertial amplification–local resonance coupled	Steel column + inclined epoxy connector	Lab	5.3–13 Hz (Rayleigh)	T −20 dB (99%) at 12 Hz; El Centro acc. −74%/−85%	Epoxy connector failure risk; Love waves unverified
Split-type pseudo-surface wave attenuating	Fan-shaped concrete + cross-shaped above-ground	Lab	1.58–14.55 Hz (Rayleigh/pseudo-SW)	T −54 dB (99.997%) at 12 Hz; Taft acc. −79%	Body waves unverified; polystyrene aging; single soil type
Subwavelength rainbow trapping	Polymer column + steel mass block	Lab	7.5–9.85 Hz (Rayleigh)	η −40 dB (99.99%) at 7.5 Hz; Northridge PGA −75%	Only clay verified; polymer–clay interface failure risk
Granular medium surface resonance	3D printed resonators in glass beads	Lab	~4.1 Hz (guided waves)	−20 dB (90%) at 200–650 Hz; range 850→200 mm	Scaling deviation; actual soil unverified
Multi-layer resonance granular medium	1–3 layer resonator arrays in glass beads	Lab	4.1–5 Hz (P-SV guided)	90% att. at 410 Hz; three-layer range reduction 80%	Particle nonlinearity unsimulated; shell corrosion risk
Three-component T-shaped core + boom	Core + boom unit (<4.5 kg)	Field	11–60 Hz (Rayleigh)	TS −20 dB (90%) at 19 Hz; PGA −60% (El Centro)	Love waves unverified; connection corrosion risk
Steel–concrete composite pile	Steel–concrete pile (D = 0.8 m, embedment 13 m)	Field	1.5–10 Hz (Rayleigh/body S-waves)	TS −15 dB (82%) at 3 Hz; PGA −87% (Kobe)	13 m embedment requires excavation; steel corrosion risk

**Table 7:** Structural vibration/energy absorption.

Type	Frequency	Attenuation/Absorption	Features
Cantilever resonance	17.49–18.13 Hz	98%	All-metal, durable
Dual-helix inertial	30–53 Hz	78%	Low frequency, tunable
Auxetic tube	Impact	7.18 J/g	High energy absorption

**Table 8:** Performance and practical attributes of BSMs, ASMs, and PESMs.

Type	Representative Attenuation	Key Advantages	Main Limitations
BSMs	Up to 99.99%	Superior attenuation, stable performance	Deep excavation, high construction cost
ASMs	93–99% (Rayleigh)	Excavation-free, cost-effective, retrofit-friendly	Higher dominant frequencies, limited control
PESMs	80–98%	Balanced performance, shallow embedment	Sensitivity to soil uniformity

## Data Availability

No new data were created or analyzed in this study. Data sharing is not applicable to this article.

## Abbreviations

The following abbreviations are used in this manuscript:

SMs	Seismic metamaterials
BSMs	Buried seismic metamaterials
ASMs	Above-surface seismic metamaterials
PESMs	Partially embedded seismic metamaterials
PGA	Peak ground acceleration
FRF	Frequency response function
